# Leiomyosarcoma of the Adrenal vein: a novel approach to surgical resection

**DOI:** 10.1186/1477-7819-5-109

**Published:** 2007-10-03

**Authors:** Tracy S Wang, Idris Tolgay Ocal, Ronald R Salem, John Elefteriades, Julie A Sosa

**Affiliations:** 1 Department of Surgery, Section of Endocrine Surgery, Yale University School of Medicine, New Haven, Connecticut, USA; 2 Department of Pathology, Yale University School of Medicine, New Haven, Connecticut, USA; 3 Department of Surgery, Section of Oncologic Surgery, Yale University School of Medicine, New Haven, Connecticut, USA; 4 Department of Cardiothoracic Surgery, Yale University School of Medicine, New Haven, Connecticut, USA

## Abstract

**Background:**

Leiomyosarcomas typically originate within smooth muscle cells. Leiomyosarcomas arising from the adrenal vein are rare malignancies associated with delayed diagnosis and poor prognosis. The most common vascular site of origin is the inferior vena cava.

**Case presentation:**

This is a 64-year old woman who presented with a 13 × 6.5 × 6.6 cm heterogeneous mass arising in the region of the right adrenal gland and extending into the inferior vena cava (IVC) and the right atrium. Biochemical evaluation excluded a functional tumor of the adrenal gland, and multiple tumor markers were negative. We present the novel use of deep hypothermic circulatory arrest (DHCA) in the resection of an adrenal vein leiomyosarcoma extending into the right atrium. The patient remains free of disease ten months after surgery. DHCA afforded a bloodless operative field for optimal resection of disease from within the IVC.

**Conclusion:**

The diagnosis of leiomyosarcomas of the adrenal vein is one of exclusion and involves preoperative radiological imaging and biochemical evaluation to exclude other functional tumors of the adrenal gland. Aggressive surgical resection is associated with improved survival and may be best achieved via collaboration among different surgical subspecialties.

## Background

Leiomyosarcoma is a soft-tissue tumor that differentiates from smooth muscle. Primary leiomyosarcomas of vascular origin are relatively rare and frequently arise within the IVC [1-3]. Rarely, leiomyosarcomas may arise from the renal or adrenal vein. We report the case of a leiomyosarcoma of the adrenal vein and a novel approach to surgical resection involving the use of deep hypothermic circulatory arrest (DHCA).

## Case presentation

**Figure 1:**
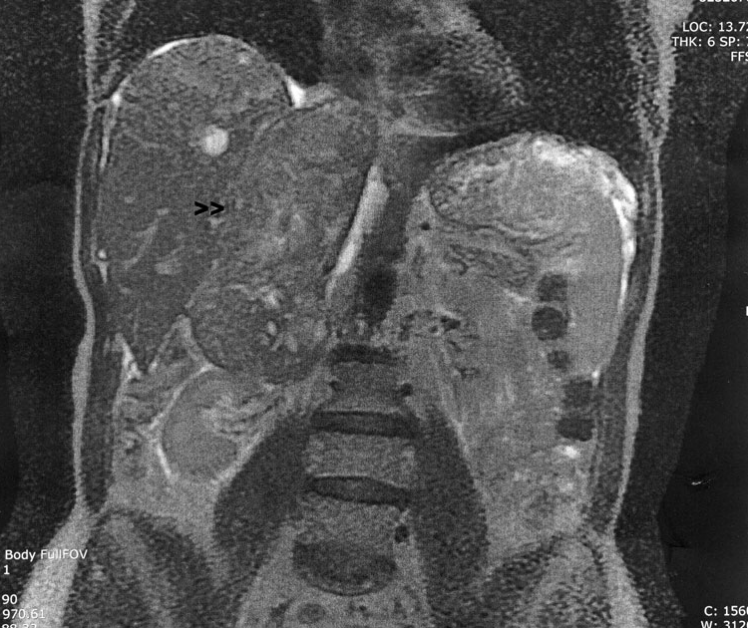
Sagittal view of abdominal MRI. Tumor (arrow) extends from the superior pole of the right kidney to the right atrium.

A 64-year old woman who presented with a 9-month history of persistent, non-productive cough, bilateral lower extremity edema, and multiple spider angiomata extending from umbilicus to ankles. The patient was otherwise healthy with no significant past medical, surgical, or family history. She denied recent weight loss, fevers, chills, nausea, vomiting, abdominal or back pain, and she had no difficulty with ambulation. Physical exam also was significant for hepatomegaly extending 4 fingerbreadths below the costal margin. There was no jaundice or virilization. Routine laboratory studies were unremarkable. Computed tomography (CT) scan revealed a 13 × 6.5 × 6.6 cm heterogeneous mass arising in the region of the right adrenal gland and extending into the IVC and the right atrium (Figure 1).

CT of the chest and magnetic resonance imaging with arterial and venous phases (MRA/MRV) revealed evidence of a filling defect within the most inferior portion of the right atrium at the confluence with the IVC. Twenty-four hour urine collections for cortisol and catecholamines were normal, as were serum aldosterone, renin, potassium, and ACTH levels. Multiple tumor markers also were normal, including dehydropepiandrosterone sulfate (DHEA-S), lactate dehydrogenase (LDH), carcinomembryonic antigen (CEA), alpha-feto protein (AFP), CA-125, and CA-19-9; only an elevated CA-125 was identified (130, normal < 35 U/mL). There was no evidence of metastases on chest CT, brain MRI, and bone scan.

The differential diagnosis included adrenocortical carcinoma, renal cell carcinoma, retroperitoneal sarcoma, or hepatoma. The patient was brought to the operating room for a planned adrenalectomy, possible right nephrectomy, cholecystectomy (for cholelithiasis), and tumor resection via IVC cavotomy under DHCA. Diagnostic laparoscopy revealed no evidence of peritoneal or hepatic metastases. Exploratory laparotomy was performed through a midline incision, followed by a Köcher maneuver. The adrenal lesion was noted to be separate from the right kidney. Intraoperative ultrasound confirmed that the tumor did not involve the caudate lobe of the liver.

**Figure 2:**
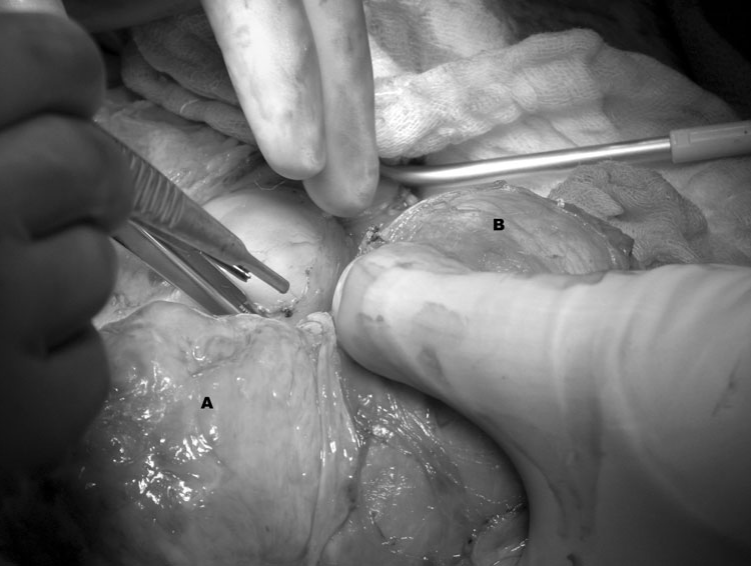
Intraoperative resection of tumor (A) from adrenal vein and IVC (B).

A median sternotomy was performed, and the diaphragm was divided in the midline anteriorly. The adrenal gland was mobilized from the liver, and the IVC was fully exposed. The patient was then placed on cardiopulmonary bypass with DHCA, and her core body temperature was lowered to 19°C. The IVC was incised, and intramural tumor was dissected free from the walls of the IVC and excised en bloc with the adrenal gland (Figure 2). There was no evidence of residual disease within the IVC or right atrium on transesophageal echocardiogram (TEE). After completion of the IVC cavotomy, the patient was taken off DHCA and re-warmed. Total circulatory arrest time was 14 minutes, with a bypass time of 115 minutes. The aorta was cross-clamped for 25 minutes. She required 6 units of packed red blood cells, 4 units of fresh frozen plasma, 4 units of platelets, and 5 liters of crystalloid. The total procedure time was 7 1/2 hours.

The patient required pharmacologic cardiovascular support for the first 12 hours postoperatively, but was awake within 24 hours. She was extubated on the morning of first postoperated day and displayed no neurocognitive deficit. The balance of her postoperative course was complicated by the development of a symptomatic pulmonary embolus, diagnosed by spiral CT of the chest. An ultrasound of the IVC also was performed to exclude tumor embolus; no evidence of thrombus was seen. She was discharged on 12th postoperative day on oral anticoagulation. Pursuant PET-CT showed no evidence of uptake in the lungs, providing further confirmation that this was a pulmonary embolus, and not tumor thrombus.

**Figure 3:**
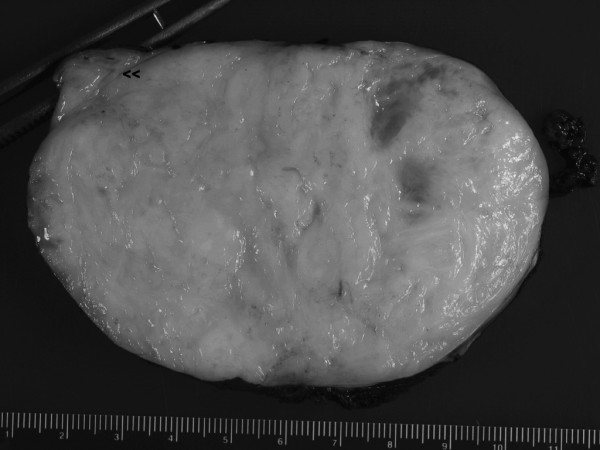
Gross specimen shows a well-circumscribed, partially encapsulated solid tumor with areas of necrosis and hemorrhage. Arrow points to normal adrenal gland.

**Figure 4:**
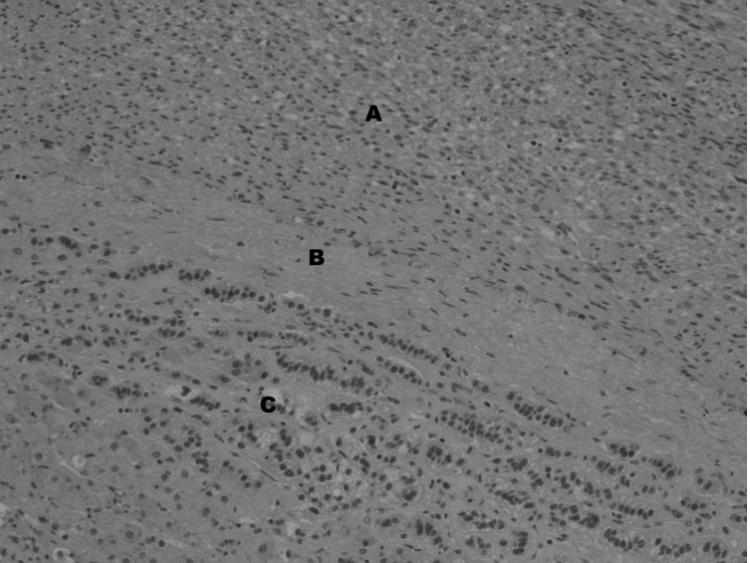
Tumor (A) shows a rim of fibrous tissue (B) separating it from adjacent adrenal cortex (C).

**Figure 5:**
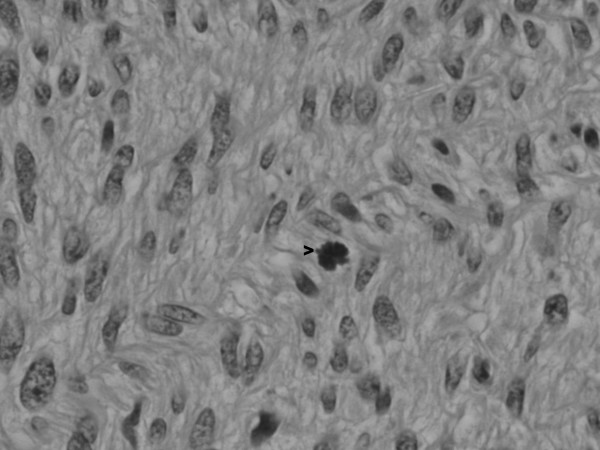
The higher power image shows spindle tumor cells with mitotic activity (arrow).

On pathological examination, the specimen measured 14.2 × 9.5 × 5.5 cm. Cross-sections of the tumor revealed a firm, nodular and trabeculated tan-white mass with focal areas of cystic degeneration and hemorrhage (Figure 3). The tumor was surrounded by a thin rim of fibrous tissue and abutted, but did not penetrate, grossly identifiable adrenal gland (Figure 4A). On microscopic evaluation, the tumor was markedly cellular, composed of spindle cells arranged in intersecting fascicles. Tumor cells showed moderate atypia with hyperchromasia, nuclear enlargement and occasional giant cells. Three to five mitoses per 10 high power field were identified, with occasional atypical mitotic figures (Figure 5). Areas of coagulative necrosis were present. Immunohistochemically, neoplastic cells were negative for c-kit, S100 and HMB45 (Dako^®^), but showed strong reactivity for desmin and smooth muscle actin (Dako^®^) (Figure 4C). This finding was consistent with leiomyosarcoma, most likely arising from adrenal vein; however, on multiple sections, no residual vascular wall was detected. Primary adrenal leiomyosarcoma was considered unlikely due to clear-cut separation of tumor from the morphologically unremarkable adrenal gland by a thin rim of fibrous tissue (Figure 4A).

## Discussion

A leiomyosarcoma of the adrenal vein was first reported in 1981, in a 50-year old patient with a 12 cm leiomyosarcoma arising from the left adrenal vein [4]. Since that time, there have been only eight other reported cases. We report a ninth case, using the novel approach of surgical resection under DHCA.

The preoperative diagnosis of a leiomyosarcoma was not established in any of the previously reported cases. However, while the exact etiology of an adrenal tumor may not be known, biochemical evaluation is warranted to exclude a functional tumor that may require preoperative medical management, i.e. alpha blockade. Excluding two historical cases identified at the time of autopsy, all tumors were ≥ 10 cm and diagnosed at the time of surgical resection, underscoring that there are no tumor markers or imaging characteristics specific to this diagnosis [3-10]. Outcome from leiomyosarcoma is poor; the longest disease-free survival is 20 months [5]. Four patients died within a year of surgery [3,7,8,10]. Our patient is currently clinically free of disease ten months after resection.

Complete surgical resection is the mainstay of treatment for adrenal vein leiomyosarcomas, as there is little evidence to support the role of chemotherapy and/or radiation. Lack *et al *reported the use of combination chemotherapy (including adriamycin) and external beam radiation to palliate bone pain in a patient with metastatic disease arising from an adrenal vein leiomyosarcoma [3]. Patient survival has been limited to 6 months following treatment with a combination of vincristine/cyclophosphamide/cisplatin/dacarbazine or external beam radiation [7,10].

Leiomyosarcoma of the adrenal vein can extend into the IVC or the right atrium [8,10]. In a report by Matsui et al, a 10 × 9 × 5 cm mass extending from between the left kidney and adrenal gland to the IVC was resected via a left radical nephrectomy-adrenalectomy with IVC thrombectomy [8]. Kato *et al *reported a right adrenal leiomyosarcoma that extended via IVC into right atrium. Tumor resection, including adrenalectomy, nephrectomy, IVC thrombectomy, and pulmonary artery thrombectomy, was approached via a midline abdominal incision, median sternotomy, and cardiopulmonary bypass [10].

To our knowledge, this is the first reported case of the use of DHCA for the resection of an adrenal vein leiomyosarcoma. The use of DHCA for resection of other retroperitoneal tumors, including renal cell carcinomas and adrenocortical carcinomas, has previously been reported [11-13]. Total circulatory arrest is only required for the critical period of tumor removal from the IVC and right atrium. Neurological sequelae of DHCA can be transient (i.e. post-operative confusion, delirium and agitation) or permanent, secondary to postoperative stroke and resulting in gross dysfunction; the upper limit of DHCA for brain protection is 40 minutes [14]. Our patient did not immediately exhibit any of these symptoms, but she developed minor disturbances in gait and difficulty with slurring of her speech six weeks postoperative. It is unclear if DHCA was related to her symptoms; CT and MRI of the head were unremarkable.

A comprehensive review of the medical literature reveals that the predominant subtype of leiomyosarcoma arises within the IVC. Still, there have been fewer than 300 reported cases since the first description by Pearl in 1871. In 1992, the International Registry of Inferior Vena Cava Leiomyosarcomas was instituted, with 218 registered patients [15]. Leiomyosarcomas may arise anywhere within the IVC. Tumor arises within the smooth muscle of the media of the IVC, and initially grows in an intramural fashion, though it ultimately extends either intraluminally and/or extraluminally. Aggressive surgical resection is the standard treatment for leiomyosarcomas of vascular origin and is associated with improved survival [2,16,17]. Overall, adjuvant treatment appears to afford no additional benefit [16,17].

## Conclusion

First-line treatment for leiomyosarcomas of the adrenal vein is radical resection with the goal of negative margins. Preoperative evaluation should include imaging to delineate the extent of disease and exclude metastases and biochemical evaluation to rule out catecholamine excess from a functional adrenocortical carcinoma or malignant pheochromocytoma requiring preoperative alpha-blockade.  Collaboration among multiple surgical subspecialties might be beneficial, and the novel role of DHCA described here should be considered in the surgical management of these tumors.

## Competing interests

The author(s) declare that they have no competing interests.

## Authors' contributions

TSW and JAS made substantial contributions to the drafting of the manuscript. RRS, JE, and TO contributed significantly to patient care. JAS was the surgeon-of-record.

All authors read and approved the final manuscript.
